# Haemin pre‐treatment augments the cardiac protection of mesenchymal stem cells by inhibiting mitochondrial fission and improving survival

**DOI:** 10.1111/jcmm.14747

**Published:** 2019-10-29

**Authors:** Rui Deng, Yaming Liu, Haiwei He, Hao Zhang, Chenling Zhao, Zhen Cui, Yimei Hong, Xin Li, Fang Lin, Dongsheng Yuan, Xiaoting Liang, Yuelin Zhang

**Affiliations:** ^1^ Department of General Surgery The Second Affiliated Hospital of Bengbu Medical College Bengbu China; ^2^ School of Pharmacy Bengbu Medical College Bengbu China; ^3^ Department of Emergency Medicine Guangdong Provincial People's Hospital Guangdong Academy of Medical Sciences Guangzhou China; ^4^ Department of Respiratory Medicine the First Affiliated Hospital of Bengbu Medical College Bengbu China; ^5^ Department of Radiation Oncology the First Affiliated Hospital of Bengbu Medical College Bengbu China; ^6^ Clinical Translational Medical Research Center Shanghai East Hospital Tongji University School of Medicine Shanghai China

**Keywords:** cell survival, haem oxygenase‐1, haemin, mesenchymal stem cells, mitochondrial fission, myocardial infarction

## Abstract

The cardiac protection of mesenchymal stem cell (MSC) transplantation for myocardial infarction (MI) is largely hampered by low cell survival. Haem oxygenase 1 (HO‐1) plays a critical role in regulation of cell survival under many stress conditions. This study aimed to investigate whether pre‐treatment with haemin, a potent HO‐1 inducer, would promote the survival of MSCs under serum deprivation and hypoxia (SD/H) and enhance the cardioprotective effects of MSCs in MI. Bone marrow (BM)‐MSCs were pretreated with or without haemin and then exposed to SD/H. The mitochondrial morphology of MSCs was determined by MitoTracker staining. BM‐MSCs and haemin‐pretreated BM‐MSCs were transplanted into the peri‐infarct region in MI mice. SD/H induced mitochondrial fragmentation, as shown by increased mitochondrial fission and apoptosis of BM‐MSCs. Pre‐treatment with haemin greatly inhibited SD/H‐induced mitochondrial fragmentation and apoptosis of BM‐MSCs. These effects were partially abrogated by knocking down HO‐1. At 4 weeks after transplantation, compared with BM‐MSCs, haemin‐pretreated BM‐MSCs had greatly improved the heart function of mice with MI. These cardioprotective effects were associated with increased cell survival, decreased cardiomyocytes apoptosis and enhanced angiogenesis. Collectively, our study identifies haemin as a regulator of MSC survival and suggests a novel strategy for improving MSC‐based therapy for MI.

## 1 INTRODUCTION

Despite the advanced developments in surgical treatment and pharmacological therapy, myocardial infarction (MI) is still a major cause of morbidity and mortality worldwide.^1^ Mesenchymal stem cell (MSC)‐based therapy has shown promising results in MI treatment because of the capacity of MSCs to differentiate into cardiomyocytes and confer paracrine effects. The efficacy of MSC‐based therapy is nonetheless seriously restricted by poor cell survival in the hostile environment of the injured heart.^2^, ^3^, ^4^ Oxidative stress in the ischaemic heart can quickly induce apoptosis of transplanted MSCs.^2^ It has been reported that fewer than 1% of MSCs can survive in the ischaemic rat heart after MI at 24 hours after transplantation.^5^ Therefore, exploring a novel strategy to enhance the retention and engraftment of MSCs in the ischaemic heart is urgently needed. Indeed, several pre‐treatment strategies, including hypoxia and genetic modification, have shown to increase the survival of MSCs under hostile environment.^6^, ^7^

Cell death is mainly mediated by mitochondrial function, which is closely related to mitochondrial dynamics.^8^ Mitochondria undergo fusion and fission to form a network for maintaining cell function.^9^, ^10^ Mitochondrial fusion is regulated by mitofusin 1 (Mfn1) and Mfn2, whereas mitochondrial fission is mainly regulated by mitochondrial fission protein dynamin‐related protein 1 (Drp1) and mitochondrial fission 1 (Fis1). Converging evidence has shown that mitochondrial fission results in fragmented mitochondria and thus induces apoptosis.^11^, ^12^ Nevertheless, whether ischaemic conditions can induce mitochondrial fission and thus lead to apoptosis of transplanted MSCs has not been determined.

Haem oxygenase 1 (HO‐1), an inducible stress protein, possesses cytoprotective defences including antioxidative stress, antiapoptosis and anti‐inflammation functions during challenge by different stressors.^13^, ^14^ A previous study has shown that HO‐1 up‐regulation inhibits mitochondrial fission, thus attenuating apoptosis of cardiomyocytes induced by intermittent hypoxia.^15^ Furthermore, cardiac‐specific overexpression of HO‐1 significantly reduces up‐regulated mitochondrial fission and therefore protects against doxorubicin‐induced dilated cardiomyopathy.^16^ Given that HO‐1 plays a critical role in regulating mitochondrial dynamics, we have been suggested that the ischaemic condition induces apoptosis of MSCs via up‐regulation of mitochondrial fission which is regulated by HO‐1. Therefore, pre‐treatment with haemin, an HO‐1 inducer, can increase the capability of MSCs to tolerate ischaemic conditions via inhibition of mitochondrial fission and thus enhance cardioprotective effects that ameliorate the damage from MI.

## 2 MATERIALS AND METHODS

### 2.1 Cell culture

Human bone marrow (BM)‐MSCs were purchased from Cambrex BioScience (catalog no. PT‐2501). BM‐MSCs were routinely cultured as previously described.^17^ Cells were passaged at a ratio of 1:3 when they reached confluence. The cells from passages 3‐4 were used in the current study.

### 2.2 Serum deprivation and hypoxia (SD/H)‐exposed cell culture and haemin pre‐treatment

To mimic the ischaemic conditions in vitro, BM‐MSCs were cultured under SD/H challenge.^18^ In brief, when BM‐MSCs reached 70%‐80% confluence, the completed culture medium was changed to medium without foetal bovine serum (FBS) and then cultured under hypoxia (1% oxygen, 5% carbon dioxide and 94% nitrogen) for 48 hours. For haemin pre‐treatment, BM‐MSCs were cultured in complete medium with 10 µM haemin under normoxia (95% air and 5% carbon dioxide) for 24 hours prior to SD/H challenge.

### 2.3 Cell‐counting kit‐8 assay

Cell viability of BM‐MSCs was examined by cell‐counting kit‐8 (CCK‐8) kit (Beyotime Biotechnology) according to the manufacturer's protocol. Briefly, BM‐MSCs with or without haemin pre‐treatment were seeded in 96‐well plates at a density of 2 × 10^3^ cells/well under SD/H challenge for 48 hours. The cells were cultured with CCK‐8 solution for an additional 2 hours at 37°C in a dark place. Subsequently, the OD values were measured at 450 nm with a microplate reader (Biotek).

### 2.4 siRNA transfection

Control siRNA or HO‐1 siRNA was used to transfect BM‐MSCs using Lipofectamine RNAiMAX (13778‐075; Invitrogen). Briefly, control siRNA or HO‐1 siRNA was diluted with OptiMEM and mixed with the transfection reagent. Each mixture was added to BM‐MSCs at 70%‐80% confluence and then incubated for 24‐48 hours. Finally, the transfection efficiency was examined by Western blot analysis.

### 2.5 MitoTracker staining

The morphology of mitochondria was examined by MitoTracker staining as previously reported.^15^ Briefly, after the different treatments, BM‐MSCs were washed with PBS and incubated with 0.01 µmol/L MitoTracker Green FM (M7514; Thermo Fisher Scientific) for 30 minutes at 37°C in a dark place and then mounted with 4',6‐diamidino‐2‐phenylindole (DAPI). Images from five different view fields of each slide (magnification of 20x) were randomly captured by laser confocal scanning microscopy (Zeiss LSM Meta 510). The percentage of mitochondrial fragmentation was calculated by comparing the number of cells with fragmented mitochondria to the total number of cells.

### 2.6 TUNEL staining

Apoptosis of BM‐MSC after different treatments was detected by terminal deoxynucleotidyl transferase‐mediated dUTP nick end labelling (TUNEL) staining kit (11684795910; Roche). Briefly, after different treatments, the cells were washed with PBS, fixed and incubated with 1 µg/mL of Proteinase K/10 mmol/L Tris solution for 15 minutes at room temperature. Following washing with PBS twice, the cells were incubated with the TUNEL reaction mixture for 1 hour at 37°C in a dark place. Finally, the cells were washed and mounted with DAPI to stain the nuclei. Images of five different view fields for each slide were randomly captured (magnification of 20x). The apoptosis of BM‐MSCs was calculated as the proportion of positive TUNEL cells to total DAPI‐positive cells.

### 2.7 Western blot analysis

The protein of each sample was extracted using RIPA buffer (9806, CST), and then the amount of concentrated protein was measured. The proteins were separated on SDS‐PAGE gel, transferred to PVDF membranes and then washed with Tris‐buffered saline (TBS) with 0.1% Tween‐20 (TBST). After blocking with 5% fat‐free milk in TBS, the membranes were incubated with the following primary antibodies: anti‐p‐Drp1 ser616 (PA5‐64821; Invitrogen), anti‐Drp1 (PA5‐20176; Invitrogen), anti‐Mfn2 (ab124773; Abcam) and GAPDH (2118, CST) at 4°C overnight. Then, the membranes were incubated with horseradish peroxide‐conjugated secondary antibodies for 1 hour at room temperature. Finally, the membranes were exposed using enhanced chemiluminescence (ECL plus) (Amersham).

### 2.8 Preparation of conditioned medium and HUVEC tube formation analysis

The conditioned medium (CdM) of MSCs was collected as previously described.^19^ Briefly, BM‐MSCs with or without haemin pre‐treatment were seeded in 6‐well plated and cultured until 70%‐80% confluence. Subsequently, the medium was replaced with 2 mL per well serum‐free medium. After 48 hours culture, the CdM was collected, centrifuged and stored at −80°C until use. HUVECs (30 000 cells/well) were seeded in a 96‐well plate coated with growth‐factor‐reduced matrigel (BD Biosciences, 356230). Next, HUVECs were treated with CdM derived from BM‐MSCs and haemin‐BM‐MSCs. After 6 hours of treatment, capillary‐like tube formation was imaged (magnification of 10x). The endothelial tube length and branching points were analysed using ImageJ software. The experiments were repeated at least three times.

### 2.9 MI model and MSC transplantation

All experiments involving animals were performed in accordance with relevant guidelines and regulations of Tongji University and approved by the Institutional Animal Care and Use Committee of the Tongji University for Laboratory Animal Medicine (TJLAC‐019‐133). Female C57/B6J mice, 6‐8 weeks old, were purchased from the Shanghai Laboratory Animal Research Center (Shanghai, China). An MI model in mice was developed as previously described.^20^ After ligation of the left anterior descending artery (LAD) 2 mm from the aorta, randomly chosen mice received an intramyocardial injection of 30 µL of PBS (MI group, n = 15), 3.0 × 10^5^ BM‐MSCs in 30 μL PBS (BM‐MSC group, n = 13) or 3.0 × 10^5^ haemin‐pretreated BM‐MSCs in 30 μL PBS (haemin‐BM‐MSC group, n = 12) at four sites on the surrounding border of the infarct area. In the negative control group (sham group, n = 10), the mice underwent thoracotomy without LAD ligation.

### 2.10 Echocardiography assessment

The heart function of each mouse from the different groups was evaluated by transthoracic echocardiography (Ultramark 9; Soma TechnologyA) at 4 weeks after cell transplantation. The echocardiographic parameters were analysed using MATLAB R2011b software (MathWorks).

### 2.11 Masson's trichrome staining

After echocardiography evaluation, all mice were killed, and the hearts were collected. The mouse hearts were fixed, embedded and sectioned into 5 μm sections. Fibrosis in the mouse hearts was detected by Masson's Trichrome Stain Kit (HT15; Sigma). Images of each slide were captured (magnification of 4x). The percentage of the infarct size was analysed as follows: (fibrosis area/total left ventricle area)×100%.

### 2.12 Immunohistochemistry

Immunohistochemical staining was performed as previously described.^3^ Briefly, the heart sections were hydrated, the antigen was retrieved, and the specimen was blocked with 5% bovine serum albumin for 30 minutes. Subsequently, heart sections were stained with the following primary antibodies, anti‐HNA (ab191181, Abcam) and anti‐CD31 (77 699, CST), at a 1:100 dilution and then incubated overnight at 4°C. After washing, the slides were incubated for 30 minutes with streptavidin peroxidase‐conjugated secondary antibody (ab64264, Abcam) at room temperature. After this incubation, the slides were washed three times in PBS, and the antibody complexes were coloured with diaminobenzidine and then counterstained with haematoxylin. Five sections were randomly collected from each mouse, and six mice from each group were captured (magnification of 10x).

### 2.13 Polymerase chain reaction

Human Alu‐sx repeat sequences in the heart tissue from the different groups were evaluated by genomic polymerase chain reaction (PCR) as previously described.^3^ The primer of human Alu‐sx was F:5'‐GGCGCGGTGGCTCACG‐3', R:5'‐TTTTTTGAGACGGAGTCTCGCTC‐3. The product was detected by electrophoresis in 1.5% agarose gel supplemented with ethidium bromide.

### 2.14 Statistical analysis

Values are shown as the mean ± SEM. Statistical analyses were performed using Prism 5.04 software (GraphPad Software Inc.). The comparison between two groups was analysed using unpaired Student's *t* tests and between multiple groups using one‐way ANOVA followed by the Bonferroni test. A *P* value <0.05 was considered statistically significant.

## 3 RESULTS

### 3.1 Haemin suppresses SD/H‐induced mitochondrial fission and apoptosis of BM‐MSCs

**Figure 1:**
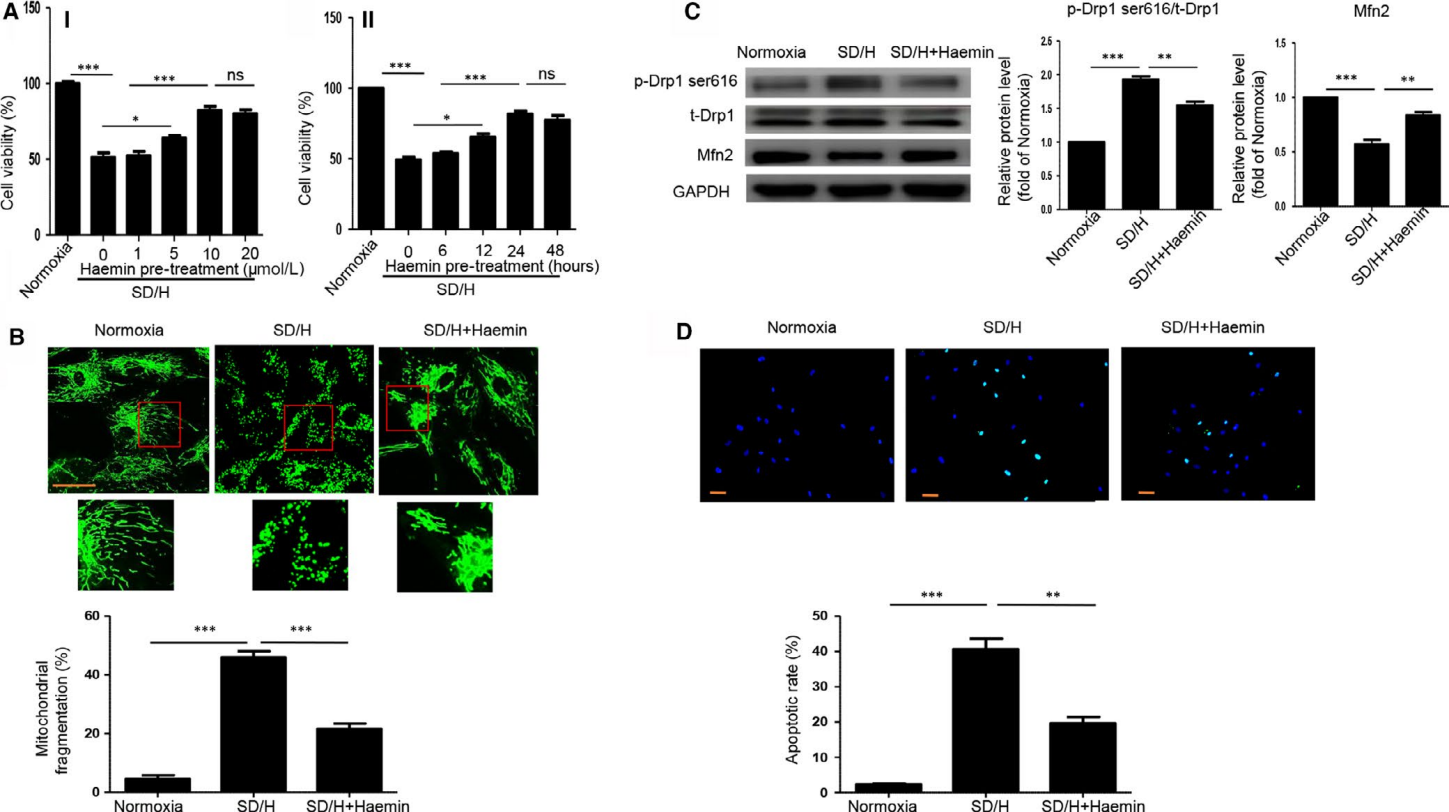
Haemin pre‐treatment suppresses serum deprivation and hypoxia (SD/H)‐induced mitochondrial fission and apoptosis of bone marrow‐mesenchymal stem cell (BM‐MSCs). A, The cell viability of BM‐MSCs with or without haemin (1, 5, 10, 20 μmol/L) pre‐treatment for 24 hours under normoxia or SD/H was determined by CCK‐8 assay (i). The cell viability of BM‐MSCs with or without 10 μmol/L haemin pre‐treatment for 6, 12, 24 or 48 hours under normoxia or SD/H was determined by CCK‐8 assay (ii). B, Representative images of the fragmented mitochondria (magnification of 20x) and quantitative analysis of fragmented mitochondria in BM‐MSCs and haemin‐pretreated BM‐MSCs under normoxia or SD/H. C, Western blotting and quantitative analysis for the expression of Mfn2 and p‐Drp1 ser616 in BM‐MSCs and haemin‐pretreated BM‐MSCs under normoxia or SD/H exposure. D, Representative images of TUNEL staining (magnification of 20x) and quantitative analysis of the apoptosis of BM‐MSCs or haemin‐pretreated BM‐MSCs under normoxia or SD/H. Data are expressed as the mean ± SEM. n = 3. Scale bar = 50 μm. *P < .05, **P < .01, ***P < .001. ns, not significant

To test the protective effects of haemin on BM‐MSCs, we pretreated BM‐MSCs with different concentration of haemin (1, 5, 10, 20 μmol/L) for 24 hours and then exposed them to SD/H. The CCK‐8 assay showed that haemin pre‐treatment greatly enhanced the viability of BM‐MSCs under SD/H in a dose‐dependent manner and 10 μmol/L haemin pre‐treatment exhibited the best protective effects (Figure 1A). Furthermore, we pretreated BM‐MSCs with 10 μmol/L haemin with different time (6, 12, 24, 48 hours) and then exposed them to SD/H. The CCK‐8 assay also showed that haemin pre‐treatment greatly enhanced the viability of BM‐MSCs under SD/H in a time‐dependent manner and 24 hours haemin pre‐treatment exerted the best protective effects (Figure 1A). Based on these results, 24 hours pre‐treatment with 10 μmol/L haemin was chosen for further studies. We then tested whether haemin pre‐treatment could regulate SD/H‐induced mitochondrial fragmentation in BM‐MSCs. The results showed that haemin pre‐treatment significantly reduced SD/H‐induced mitochondrial fragmentation in BM‐MSCs (Figure 1B). Western blotting demonstrated that haemin pre‐treatment reversed the up‐regulation of p‐Drp1 ser616 and the down‐regulation of Mfn2 induced by SD/H in BM‐MSCs, suggesting that haemin attenuated SD/H‐induced mitochondrial fission in BM‐MSCs (Figure 1C). Moreover, haemin pre‐treatment ameliorated SD/H‐induced apoptosis of BM‐MSCs (Figure 1D). Taken together, these findings indicate that haemin suppresses SD/H‐induced mitochondrial fission and apoptosis of BM‐MSCs.

### 3.2 Haemin inhibits mitochondrial fragmentation and apoptosis of BM‐MSCs by regulating HO‐1

**Figure 2:**
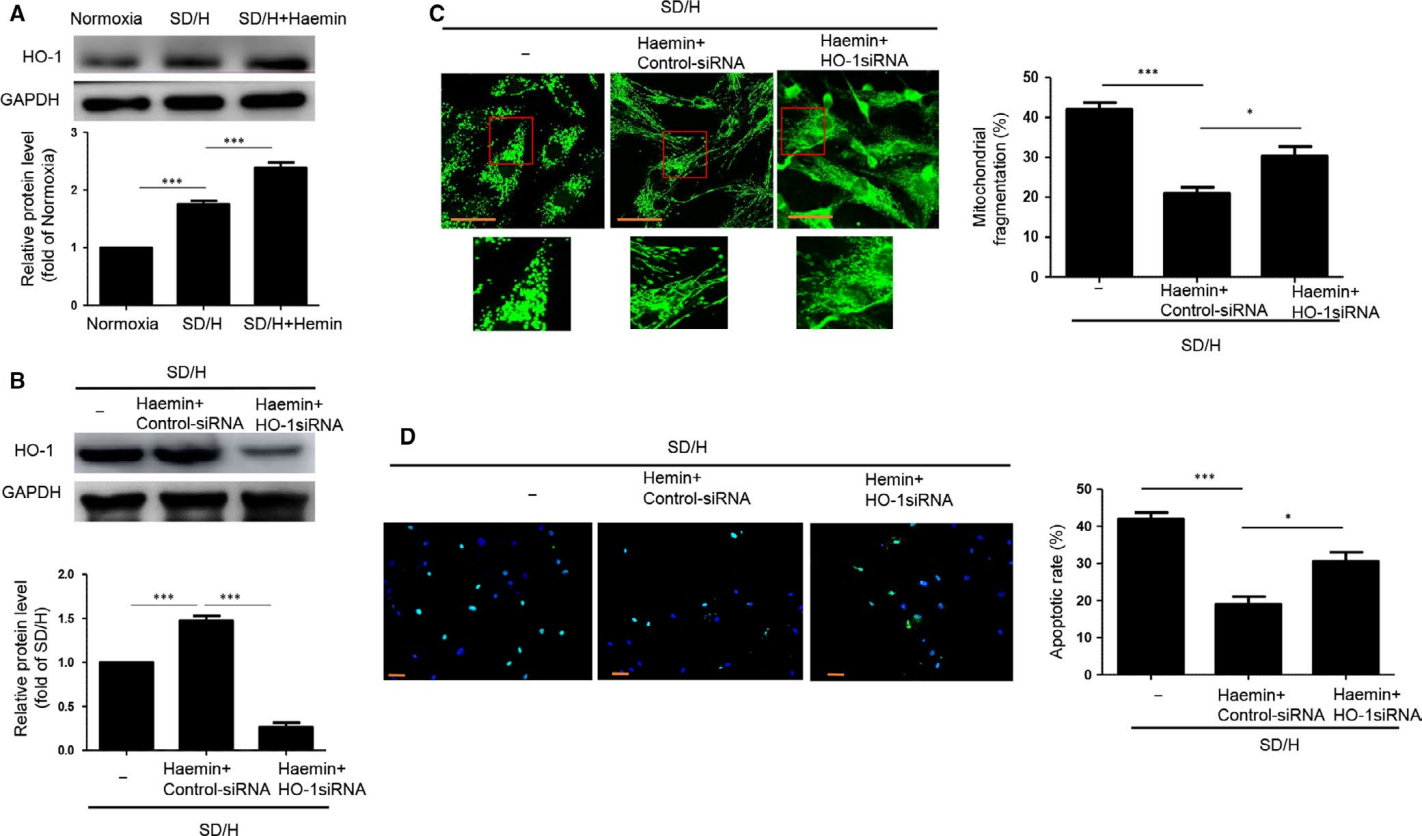
Haemin pre‐treatment inhibits mitochondrial fragmentation and apoptosis of bone marrow‐mesenchymal stem cell (BM‐MSCs) by regulating HO‐1. A, Western blotting and quantitative analysis for the expression of HO‐1 in BM‐MSCs or haemin‐pretreated BM‐MSCs under normoxia or SD/H. B, Western blotting and quantitative analysis for the expression of HO‐1 in BM‐MSCs and haemin‐pretreated BM‐MSCs treated with control siRNA or HO‐1 siRNA under SD/H. C, Representative images of the fragmented mitochondria (magnification of 20x) and quantitative analysis of fragmented mitochondria in BM‐MSCs and haemin‐pretreated BM‐MSCs treated with control siRNA or HO‐1 siRNA under SD/H. D, Representative images of TUNEL staining (magnification of 20x) and quantitative analysis of the apoptosis of BM‐MSCs and haemin‐pretreated BM‐MSCs treated with control siRNA or HO‐1 siRNA under SD/H. Data are expressed as the mean ± SEM. n = 3. Scale bar = 50 μm *P < .05, ***P < .001

As haemin is an HO‐1 inducer, we investigated whether the protective effects of haemin on SD/H‐induced BM‐MSCs after injury are the result of haemin regulation of HO‐1. Western blotting showed that SD/H enhanced the expression of HO‐1 in BM‐MSCs, and haemin pre‐treatment further increased the expression of HO‐1 in BM‐MSCs under SD/H, indicating that the protective effects of haemin may be involved in the regulation of HO‐1 (Figure 2A). To verify whether the protective effects of haemin in BM‐MSCs under SD/H were caused by regulation of HO‐1, we added HO‐1 siRNA to the haemin‐pretreated BM‐MSCs and then exposed the cells to SD/H. The elevation of HO‐1 expression induced by haemin under SD/H was significantly abolished in the haemin‐pretreated BM‐MSCs with HO‐1 siRNA (Figure 2B). Notably, mitochondria fragmentation and cell apoptosis were significantly increased in the haemin‐pretreated BM‐MSCs with HO‐1 siRNA compared with haemin‐pretreated BM‐MSCs under SD/H (Figure 2C,D). Taken together, these results show that haemin inhibits mitochondrial fragmentation and apoptosis of BM‐MSCs induced by SD/H conditions through activating HO‐1.

### 3.3 Haemin‐pretreated BM‐MSCs enhance cardioprotection following MI in mice

**Figure 3:**
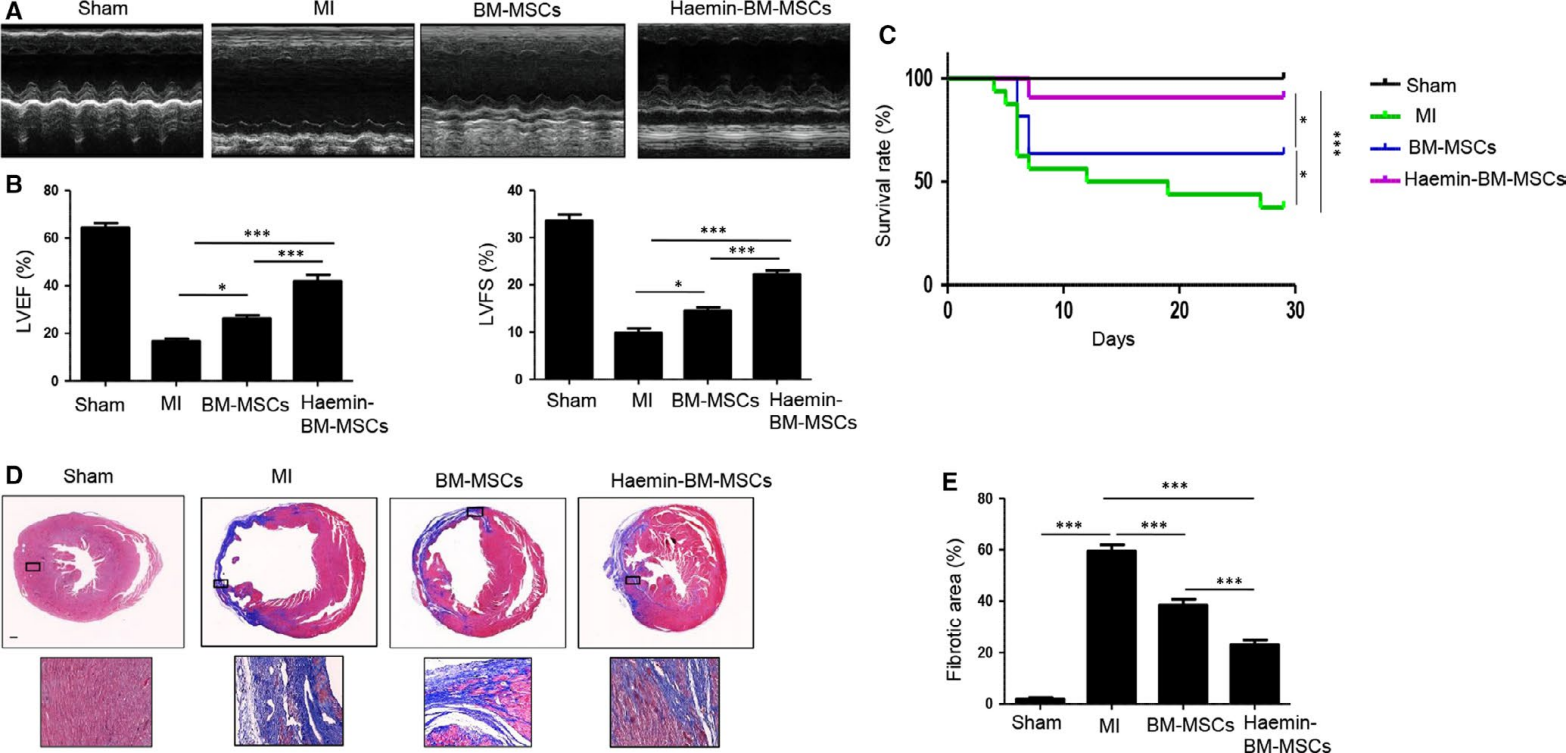
Haemin pre‐treatment enhances BM‐MSC‐mediated cardioprotection following infarction. A, Representative images of M‐mode echocardiographic images captured at 4 weeks after MI in mice among different groups. B, Quantitative analysis of LVEF and LVFS at 4 weeks after MI in mice among the different groups. C, Kaplan‐Meier curves showed the survival rate of mice in the different groups. D, Representative images of Masson's trichrome staining of heart sections at 4 weeks after MI in mice among the different groups (magnification of 4x). E, Quantitative measurement of heart fibrosis at 4 weeks after MI in mice among the different groups. Data are expressed as the mean ± SEM. n = 6. Scale bar = 200 μm. *P < .05, **P < .01, ***P < .001

Cardiac function was measured by echocardiography. Representative images of M‐mode echocardiography were captured 4 weeks after MI in mice (Figure 3A). Transthoracic echocardiography showed that left ventricle ejection fraction (LVEF) and fraction shortening (LVFS) were significantly reduced in the MI group compared with the sham group (Figure 3B). Both MSC‐transplanted groups showed a significant increase in LVEF and LVFS, and LVEF and LVFS were significantly greater in the haemin‐BM‐MSC group than in the BM‐MSC group (Figure 3B). Next, we analysed the survival rate of mice from the different groups. As shown in Figure 3(C), compared with the sham group, the mortality of mice was greatly increased in the MI group. Nevertheless, the mortality rate of mice in the BM‐MSCs group and haemin‐BM‐MSCs group was significantly decreased compared with the MI group. Notably, the mortality rate in the haemin‐BM‐MSCs group was much lower than BM‐MSCs group (Figure 3C). Masson's trichrome staining showed that interstitial fibrosis was greatly enhanced in the MI groups compared with the sham group (Figure 3D,E). MSC transplantation decreased the infarct area, and the haemin‐BM‐MSC group exhibited a significantly decreased infarct area compared with the BM‐MSC group (Figure 3D,E).

### 3.4 Haemin‐pretreated BM‐MSCs improved cell survival in mouse hearts following MI

**Figure 4:**
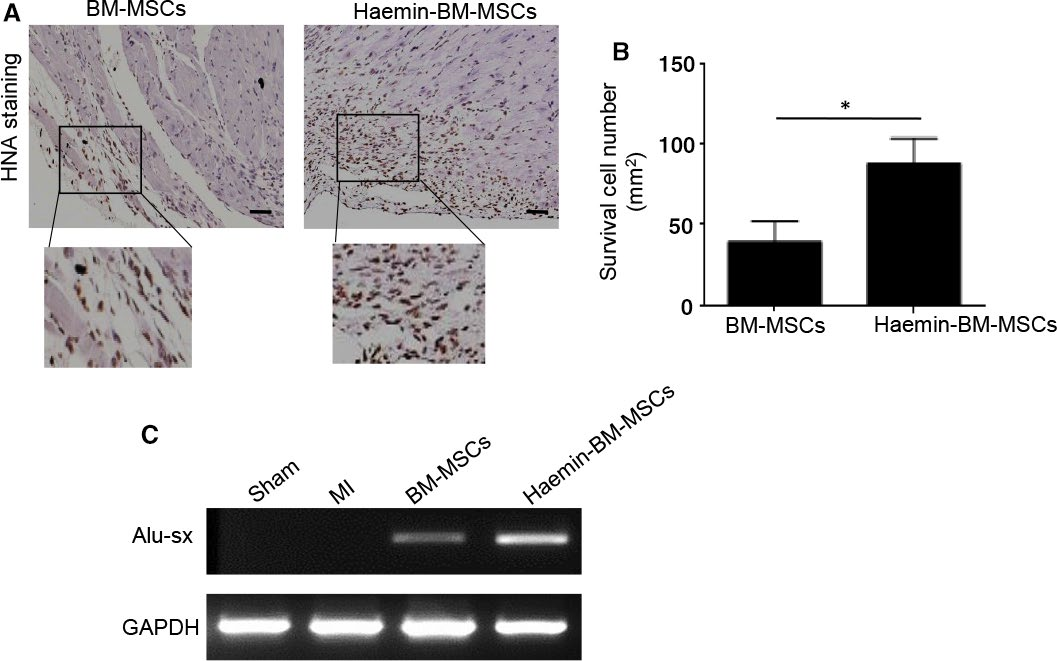
Haemin‐pretreated bone marrow‐mesenchymal stem cell (BM‐MSCs) improved cell survival in mouse hearts following MI. A, Representative images of anti‐HNA‐positive cells in the ischaemic heart at 4 weeks after cell transplantation (magnification of 10x). B, Quantitative analysis of cell survival at 4 weeks after MI in mice among the different groups. C, PCR showed that the expression of Alu‐sx was significantly increased in the haemin‐BM‐MSCs group compared with the BM‐MSCs group. Data are expressed as the mean ± SEM. n = 6. Scale bar = 100 μm. *P < .05

We first performed anti‐HNA staining to detect cell survival at 4 weeks after transplantation. Both BM‐MSCs and haemin‐pretreated BM‐MSCs were detected in ischaemic heart tissue, with a much higher cell survival detected for the haemin‐BM‐MSC group, suggesting that haemin improves BM‐MSC tolerance against ischaemia challenge (Figure 4A,B). To further verify the survival of MSCs in ischaemic heart tissue after transplantation, we performed PCR to detect the human repeat sequences Alu‐sx in heart tissue from different groups. Alu‐sx was detected in the BM‐MSCs group and haemin‐BM‐MSCs group, but not in the sham group and MI group (Figure 4C). Furthermore, the expression of Alu‐sx was significantly increased in the haemin‐BM‐MSCs group compared with the BM‐MSCs group (Figure 4C). Taken together, these data demonstrated that haemin treatment promotes BM‐MSC survival in the ischaemic heart tissue.

### 3.5 Haemin‐pretreated BM‐MSCs inhibited the apoptosis of cardiomyocytes and improved angiogenesis in mouse hearts following MI

**Figure 5:**
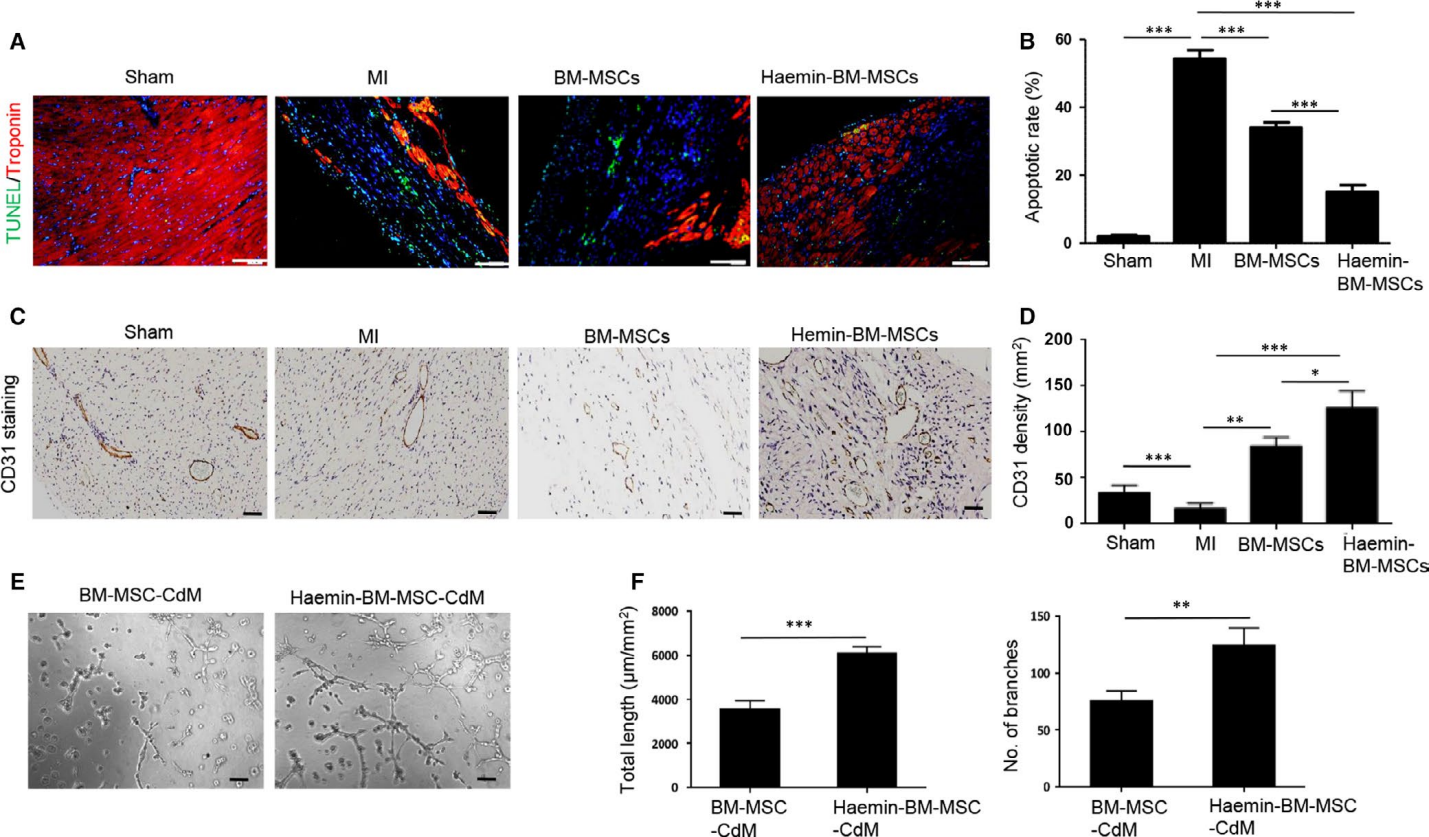
Haemin‐pretreated bone marrow‐mesenchymal stem cell (BM‐MSCs) improved angiogenesis in mouse hearts following MI. A, Representative images of TUNEL staining in the heart at 4 weeks among the different groups (magnification of 20x). B, Quantitative analysis of the apoptosis of cardiomyocytes in the heart at 4 weeks among the different groups. n = 6. C, Representative images of CD31 staining in the heart at 4 weeks among the different groups (magnification of 10x). D, Quantitative analysis of blood vessels in the border zone of ischaemic hearts stained with CD31. n = 6. E, Representative light images of HUVEC tube formation assay in BM‐MSC‐CdM and haemin‐BM‐MSC‐CdM treatment (magnification of 10x). F, Quantitative analysis of HUVEC tube length and number of the branches in BM‐MSC‐CdM and haemin‐BM‐MSC‐CdM treatment. n = 3. Data are expressed as the mean ± SEM. *P < .05, **P < .01, ***P < .001*.* Scale bar = 100 μm

The apoptosis of cardiomyocytes among the different groups was assessed by TUNEL staining. Compared with the sham group, the apoptosis of cardiomyocytes was dramatically increased in the MI group (Figure 5A,B). MSC transplantation greatly inhibited the apoptosis of cardiomyocytes, and haemin‐BM‐MSCs were superior to BM‐MSCs in attenuating the apoptosis of cardiomyocytes in the ischaemic hearts of mice (Figure 5A,B). The capillary density of the ischaemic area among the different groups was detected by CD31 staining. The capillary density was decreased in the MI group compared with the sham group (Figure 5C,D). The capillary density of the ischaemic area increased following MSC treatment (Figure 5C,D). Notably, the haemin‐BM‐MSC group had a much higher capillary density than the BM‐MSC group (Figure 5C,D). To further examine the paracrine effects of MSCs, the angiogenic capacity of CdM derived from BM‐MSCs and haemin‐BM‐MSCs was assessed by the capillary tube formation assay. Compared with BM‐MSC‐CdM, the endothelial tube length and branching points were significantly increased in the haemin‐BM‐MSCs‐treated HUVECs, demonstrating increased endothelial network formation (Figure 5E,F). Collectively, these data demonstrated haemin‐pretreated BM‐MSCs inhibited the apoptosis of cardiomyocytes and improved angiogenesis in mouse hearts following MI.

## 4 DISCUSSION

**Figure 6:**
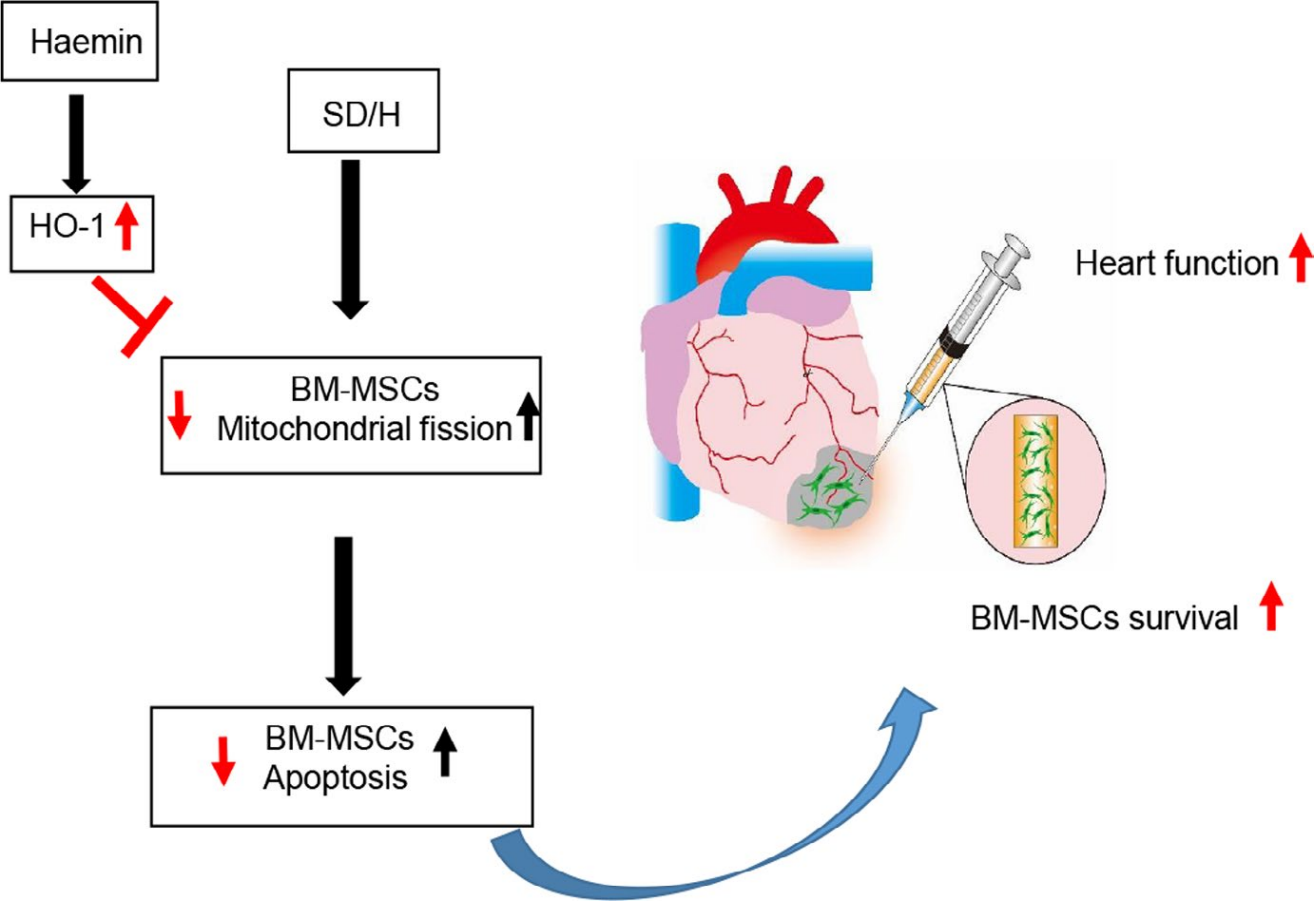
Transplantation of haemin‐pretreated BM‐MSCs dramatically improves heart function recovery after MI in mice via enhancement of cell survival

This study presents several major findings (Figure 6). First, haemin pre‐treatment improved the survival of BM‐MSCs under SD/H challenge. Second, haemin pre‐treatment inhibited SD/H‐induced mitochondrial fission and apoptosis of BM‐MSCs. Third, haemin pre‐treatment significantly increased the engraftment of BM‐MSCs and induced angiogenesis in a mouse model of MI and thus enhanced cardiac protection efficacy.

MI is a major contributor to the mobility and mortality of people with cardiovascular diseases, accounting for 11.2% of deaths worldwide.^21^ The ischaemic condition caused by insufficient blood flow leads to a marked loss of cardiomyocytes in the heart. Furthermore, the injured heart cannot compensate for the lost cardiomyocytes because of limited regenerative ability, leading to cardiac remodelling. Over the past decades, stem cell‐based therapy has emerged as a novel strategy for treating MI. Among the various types of stem cells currently used in pre‐clinical and clinical trials, MSCs have been considered a favourable cell source for MI treatment because of their unique advantages, such as easy isolation, low immunogenicity and multipotent differentiation capacity.^20^, ^22^ However, most MSCs died within 3 days after transplantation in the harsh environment of the injured heart, thus limiting therapeutic efficacy.^23^ Recently, accumulating evidence has demonstrated pharmacological pre‐treatment as a novel strategy to promote the survival of transplanted BM‐MSCs in the ischaemic heart.^24^, ^25^ Pre‐treatment with sphingolipid metabolite sphingosine 1‐phosphate dramatically enhanced the engraftment of MSCs and thus improved the therapeutic efficacy in a mouse model of MI.^26^ Pre‐treatment with fucoidan inhibited H_2_O_2_‐induced apoptosis of BM‐MSCs by regulating the MAPK and Akt signalling pathways. Furthermore, transplantation of fucoidan‐pretreated MSCs functionally attenuated limb salvage in a murine hindlimb ischaemia model, and these protective effects were attributed to enhanced cell survival.^27^ Given these findings, identifying a novel drug or biological factor to pretreat BM‐MSCs prior to transplantation is of great importance.

HO‐1, one isoform of the HO enzyme system, is expressed in many types of cells and can be induced under the pathophysiological conditions.^13^ The elevation of HO‐1 exhibits cellular protection via various mechanisms. Previous studies have shown that HO‐1 plays a critical role in regulating cell survival in response to pathophysiologic stimuli. Activation of HO‐1 enhanced the survival of cardiomyocytes under intermittent hypoxia challenge, whereas knocking down HO‐1 partially abrogated this effect.^15^ Indeed, transduction of HO‐1 into BM‐MSCs can improve cell survival under stress conditions. MSCs overexpressing HO‐1 exhibited superior prosurvival and antiapoptotic properties and thus exerted an enhanced protective efficacy to attenuate lipopolysaccharide‐induced acute lung injury in rats.^28^ Transfection with HO‐1 in MSCs greatly attenuated iron overload‐induced apoptosis by inhibiting reactive oxygen species generation and IL‐10 secretion.^29^ Nevertheless, gene modification can affect the genome stability of MSCs. Therefore, in the current study, we used haemin pre‐treatment to induce HO‐1 expression in BM‐MSCs. Consistent with previous studies, pre‐treatment with haemin significantly enhanced the expression of HO‐1 in BM‐MSCs under SD/H, and the elevation of HO‐1 greatly inhibited apoptosis of BM‐MSCs. More importantly, HO‐1 knockdown with siRNA remarkably reversed the antiapoptotic effects in BM‐MSCs. In vivo, haemin pre‐treatment significantly enhanced the engraftment of BM‐MSCs and restored heart function in a mouse model of MI compared with BM‐MSCs without haemin pre‐treatment. Although haemin pre‐treatment can increase the survival of BM‐MSCs under SD/H challenge, the underlying mechanisms remain largely unknown.

Mitochondria dynamics play an essential role in inducing cell death.^30^ Mitochondrial fusion leads to elongated mitochondria, whereas mitochondrial fission produces small round mitochondria.^10^ There is a balance of mitochondrial fusion and fission in a healthy cell. However, this balance is disrupted under stress conditions, resulting in apoptosis.^31^ In the current study, we found that the mitochondrial fragmentation of BM‐MSCs was dramatically increased under SD/H challenge compared with normoxia. Furthermore, SD/H challenge up‐regulated the expression of p‐Drp1 ser616 and down‐regulated Mfn2, indicating SD/H induced mitochondrial fission. Recent studies have documented that HO‐1 plays an essential role in the regulation of mitochondrial fission.^32^, ^33^ Haemin treatment significantly inhibited the ischaemia/reperfusion‐induced up‐regulation of Drp1 expression and enhanced the down‐regulation of Mfn2 in a mouse model of liver injury and thus attenuated hepatic injury. Furthermore, these protective effects were partially reversed by ZnPP, a haemin inhibitor.^34^ Consistently, in this study, we found that haemin pre‐treatment enhanced the expression of HO‐1 in BM‐MSCs and ameliorated mitochondrial fission, as shown by reduced Drp1 levels and increased Mfn2 levels. More importantly, inhibition of HO‐1 through siRNA addition greatly abrogated the inhibition of haemin on mitochondrial fission and apoptosis. However, whether chemical inhibition of HO‐1 activity using ZnPP affects mitochondrial fragmentation and apoptosis requires further investigation.

This study has several limitations. First, in addition to Drp1 and Mfn2, whether haemin can affect other proteins related to mitochondrial dynamics has not been determined. Second, we only examined the survival of haemin‐pretreated BM‐MSCs at 4 weeks after transplantation; therefore, long‐term cell survival needs to be examined in future studies. Third, the potential mechanisms behind HO‐1 regulation of mitochondrial dynamics remain unclear. Haemin contains iron, which is released by HO activity, regulating the expression of various proteins. As mitochondria are the major iron handling organelles, whether haemin regulates mitochondrial dynamics via iron requires further investigation. Fourth, as SD/H enhances endogenous HO‐1 expression level, it therefore would make scientific sense to silence basal HO‐1 levels to verify our study.

In summary, our results demonstrated that haemin pre‐treatment, via up‐regulation of HO‐1 levels, significantly enhanced BM‐MSC survival under ischaemic conditions by inhibiting mitochondrial fission, thus improving the therapeutic effects for treating MI. Our study shows pharmacological pre‐treatment modulating the HO‐1 pathway as a novel approach for enhancing MSC‐based therapy for cardiovascular diseases.

## CONFLICT OF INTEREST

The authors declare no conflicts of interest.

## AUTHOR CONTRIBUTIONS

Xiaoting Liang and Yuelin Zhang designed experimental, analysed data and wrote manuscript; Rui Deng, Yaming Liu and Haiwei He performed experiment, collected data and wrote manuscript; Hao Zhang, Chenling Zhao, Zhen Cui and Yimei Hong performed experiment and collected data; Xin Li analysed data and reviewed manuscript; and Fang Lin and Dongsheng Yuan interpreted the results.

## Data Availability

The data sets used and/or analysed during the current study are available from the corresponding author on reasonable request.
